# Feasibility and acceptability of a multi-component mental wellbeing program for young high-performance athletes in Australian Rules Football

**DOI:** 10.3389/fspor.2024.1470726

**Published:** 2024-11-21

**Authors:** Erin Hoare, Nicky Couston, Lilli Burdon, Claudia Vella, Kate Hall

**Affiliations:** ^1^ Australian Football League, Melbourne, VIC, Australia; ^2^ IMPACT - The Institute for Mental and Physical Health and Clinical Translation, School of Medicine, Barwon Health, Deakin University, Geelong, VIC, Australia; ^3^ School of Psychology, Deakin University, Burwood, VIC, Australia

**Keywords:** mental well-being, well-being, young high-performance sport, Australia, adolescence

## Abstract

There is a strong rationale for the promotion of mental wellbeing in elite sport development pathways although evidence-based programs are scant. Scholarly work more broadly indicates the importance of co-design and lived experience in the development of programs targeting mental health and wellbeing for young people. Further, the evidence to date suggests that programs should be multi-component to target the various systems within which young people engage. This study examines the feasibility and acceptability of a multi-component wellbeing program, informed by positive psychology and wellbeing science. The AFL Talent Pathways Wellbeing Program aims to foster positive mental health and wellbeing among young (16–18 year-old) athletes in Australian Rules Football participating in the AFL Talent Pathways. The program was comprised of multiple components including (i) a wellbeing curriculum informed by positive psychology, (ii) strengthened localized dedicated capacities in personnel within football communities, and (iii) an individualized wellbeing development plan. Feasibility and acceptability were assessed through qualitative interviews with young athletes, coaches, and wellbeing coordinators which focused on experiences related to program engagement, satisfaction, participation, and retention. A pre-specified thematic analytical approach was adopted, whereby qualitative interview data were transcribed, and then underwent a six-phase analysis process to assess themes. Results suggested gradual acceptance of the program, with increasing levels of connection and support reported throughout the program delivery. Barriers to engagement included the scheduling of the program content within the context of a comprehensive training schedule and the travel time required to be able to participate in the program particularly for non-metropolitan and regional-based athletes. Strengthening football community capacity for specific wellbeing-related needs was seen as a major facilitator to the acceptability and feasibility of the wellbeing program. These findings, although limited by a small sample size, contribute to the growing evidence body of supporting young athletes transitioning into elite sport and is strengthened through the qualitative insights achieved through the lived experience of program participants.

## 1 Introduction

The period of adolescence has been established as a particularly important time period when mental health and wellbeing can be fostered (1). This time coincides with maturation from childhood to adulthood, marked by increasing independence, and the context of onset of mental disorders – most of which emerge by early adulthood (2). Scholars and practitioners are in agreement that early life offers an important opportunity to build skills and competencies that can support healthy development into adulthood (3). Challenges remain, however, in how to best engage young people in mental health promoting initiatives, and how to sustain such efforts long term (4).

It has been established that individuals engaged in high performance sport experience a range of sport specific stressors that may pose risks to mental health and wellbeing (5). The youth athlete population is unique in that adolescents generally experience unique stressors and risk factors for mental health and wellbeing relative to children or adult populations, and this is intersected with the unique experiences that characterize high performance sport (6). There is recognition of the importance of supporting the mental health and wellbeing of young athletes, and that such support is likely to enable improved health, performance, engagement, and other outcomes. However, to date, there is little research in terms of programmatic and intervention-based research design for and responsive to, the unique needs of young elite athletes (5). A recent opinion piece reported a proposed framework for supporting young high-performance athletes transitioning into elite sport, citing multiple interacting risk factors that operate across individual, familial, sport specific, and socio-cultural levels (7). Also recognized was the dearth of evidence relating to transitioning into elite sport as opposed to transitioning into retirement (7).

The previous literature review revealed that mental wellbeing in young high performance sport has primarily been investigated as a secondary outcome to programs designed to treat, or intervene early, in mental ill health (8). Some literature was identified examining stand-alone positive psychology interventions, along with the need for a model of wellbeing to meet the needs of young high performance athletes. The wider public health and mental health evidence body suggests that discrete, stand-alone programs that are designed to solely increase skills within individuals, are unlikely to be sustained long term (9). Program outcomes are enhanced when the local contextual factors are incorporated into the design and delivery, and whereby the target participants are themselves involved in the continuous improvement processes to ensure their unique needs are understood and met (10).

As per the previous study, and briefly summarized here, the Australian Football League (AFL) is the pre-eminent, professional sporting competition of Australian rules football. The League includes both the men's and women's (AFLW) professional leagues. The AFL Talent Pathways Wellbeing Program is the AFL youth program for talent identified players aged 16–19 years in high performance leagues and Academies throughout the country. Most young players who receive the program participate in the Coates League (formerly NAB League) competition. Annually, there are approximately 1,500 athletes who participate and compete in the high-performance league, to prepare the athletes for potential drafting into the professional men's and women's competitions. As a core component of the AFL Talent Pathways Wellbeing Program, the athletes participate in a dedicated wellbeing program, further described below and in our previous published work (8). The wellbeing program is informed by positive psychology and is delivered throughout the football season to provide young people with the skills and competencies to support wellbeing in the high-performance environment.

This current study aimed to understand the acceptability and feasibility of the AFL Talent Pathways Wellbeing Program from the perspectives of the young people engaged in the program and through the experiences of the coaches and wellbeing coordinators during the 2021 season. This AFL Talent Pathways Wellbeing Program sits within a model of continuous improvement thus the findings of this study were designed to be used in the further refining and development of the program. Feasibility is defined as the extent to which an innovation can be successfully carried out within a setting and acceptability explores how the individuals involved in the intervention perceived their experience of the program (11). The evaluation of the feasibility and acceptability will demonstrate areas for improvement and development in future iterations of the wellbeing curriculum and may inform development of curricula for young elite athletes. This study received ethics approval from Deakin University Human Research Ethics Committee (HEAG-H 144_2021).

## 2 Methods

### 2.1 Programmatic elements

As per our previous work and briefly summarized here, the AFL Talent Pathways Wellbeing Program, herein the Wellbeing program or program, is designed to contribute to an environment of positive mental wellbeing (Table 1). The purpose of this is in response to the academic literature in supporting both athlete awareness and encouraging help seeking, alongside wider systemic changes such as supportive policies, to create safe, inclusive and mentally healthy systems and environments (7, 8). A major component of this model is the delivery of a wellbeing curriculum to the young athletes, informed by positive psychology PERMA model of wellbeing (12), to the young athletes. The curriculum focuses on building skills and strategies for young people to care for and build their own wellbeing through the pillars of PERMA: positive emotion, engagement, relationships, meaning, and accomplishment (12). The curriculum includes a parent education component to upskill parents and guardians in wellbeing literacy to build capacity in the home environment, and to create shared responsibility and language for young people's wellbeing. The delivery of this wellbeing curriculum occurs weekly throughout the football season and is facilitated by AFL-employed, local wellbeing co-ordinators, who hold minimum or working toward Bachelor-level qualifications in health, education, or science. Individual THRIVE wellbeing planning occurs between youth athletes and wellbeing coordinators, which aims to assist players to consider domains of their life (including but not exclusive to football) they can focus on to build wellbeing. The development of the Wellbeing Program including a literature review of evidence and underpinning theoretical foundations, is articulated in our previous publication (7). Table 1 describes an overview of the program components.

**Table 1:** Description of the components of the AFL talent pathways wellbeing program (2021).

Component of the AFL talent pathways wellbeing program (2021)	Purpose of the component
Wellbeing coordinator	Fourteen wellbeing coordinators were employed, each responsible for one region in Victoria or Tasmania, Australia. The wellbeing coordinator's role was to facilitate the development and implementation of wellbeing strategies and manage the delivery of the wellbeing curriculum. They provided individual support and guidance (THRIVE wellbeing planning) to encourage athletes to pursue and sustain good mental health and wellbeing.
THRIVE wellbeing individual development plan (THRIVE)	The wellbeing coordinators are responsible for completing the THRIVE wellbeing individual development plan with each of the talent pathway athletes in their regional program. This development plan encompasses a comprehensive understanding of the athletes’ non-athletic endeavours, levels of support, areas of vulnerability and goals for development. The outcome is to identify the athletes’ personal strengths, possible challenges, and opportunities to support the athlete in a talent development environment.
Wellbeing curriculum	The wellbeing curriculum, shown in detail in Appendix A, was created by the AFL Talent Wellbeing Team and is centred on the building blocks of wellbeing introduced in the PERMA model of wellbeing (12). The components of the curriculum have a wellbeing focus that pertain to one of the five PERMA domains: positive emotion, engagement, relationship, meaning, accomplishment. There were additional wellbeing seminars presented to the male and female athletes; “Problematic Drinking Conversation” and presented to the female athletes; ‘The High-Performance Period Webinar.’
Policies supporting wellbeing culture	AFL Managing Psychological Risk for young people policy. AFL Safeguarding Children and Wellbeing policy. AFL Respect and Responsibility Policy.

### 2.2 Study design

Throughout the 2020–2021 NAB League season all players (*n* = 608) were enrolled in the Wellbeing Program. Qualitative interviews were conducted by a student researcher (LB) with eight NAB league players (three male; three female), one coach, one AFL administrator and two wellbeing coordinators who had responded after being sent a study invitation with study information and informed consent processes by email. Eligibility criteria included being over the age of 18 years who played in the NAB league in the 2021 season. The interviews were conducted individually over a 50-minute semi-structured zoom interview. Interview questions are reported in Table 2. Participants’ written informed consent were obtained and participants were advised that the interview will be recorded for coding purposes, and that a transcript of the Zoom will be created by the student researcher (LB). Participants were advised that all identifying information will be removed from the analysis and publication and that it was not possible to attribute any of their comments to them.

**Table 2:** Interview questions.

Acceptability	
Engagement	What parts of the Wellbeing Program helped you most with your wellbeing throughout the season? How did your wellbeing coordinator impact your experiences with the Wellbeing Program? Did you feel motivated to engage in the Wellbeing Program, did your motivation change throughout the season? Did you prefer certain aspects of the Wellbeing Program delivered online or in-person?
Satisfaction	What did you like most about the Wellbeing Program (wellbeing coordinator, wellbeing curriculum and individual THRIVE sessions), and why? What did you not like about the Wellbeing Program (wellbeing coordinator, wellbeing curriculum and individual THRIVE sessions) and why? If you could change anything to the Wellbeing Program, what would you change? What could be done to make the Wellbeing Program more valuable for you and your teammates?
Feasibility	
Participation	Did you have enough time to complete the wellbeing curriculum? Did you feel confident to contribute in the group and individual sessions? What impacted your ability to participate in the group and individual sessions?
Retention	As the season progressed did you still feel the Wellbeing Program was a valuable use of your time, and involvement with the NAB League, why? Do you have suggestions for how we could adjust the schedule of the Wellbeing Program around your season?

### 2.3 Measures

A full list of questions used in the qualitative interviews is reported in Table 2. The questions were derived from existing literature related to feasibility and acceptability and adapted to meet the unique context of the AFL Talent program pathway and target population (i.e., players, coaches, wellbeing coordinators) acting as interviewees (13, 14).

### 2.4 Statistical analysis

This acceptability and feasibility study utilized a pre-existing thematic analytical approach to assess the acceptability and feasibility of the wellbeing program (15). The six-phased thematic analysis involves (i) familiarizing with the data, (ii) generating initial codes, (iii) searching for themes, (iv) reviewing themes, (v) defining and naming themes, (vi) results generation. This approach has previously been identified as a rigorous process through which data can be interpreted and represented. This process is described more comprehensively elsewhere (15) but incorporates data triangulation, reflexive journaling, hierarchies of concepts and documentation in relation to reasons for theoretical, methodological, and other choices throughout the data interpretation. The use of this data for the purpose of this current study has previously received ethics approval from Deakin University Human Research Ethics Committee (HEAG-H 144_2021). To manage the conflict of interest and as per the ethical guidelines mandated through the overseeing committee, authors who were employed by Australian Football League were restricted from accessing or contributing to the data analysis. LB led the interviews, recording and transcribing, and CV completed the six-phase thematic analysis and results summary.

## 3 Results

### 3.1 Acceptability

#### 3.1.1 Engagement

The results here relate both to engagement in the program, and also factors affecting the participants engagement. Participants expressed a sense of gradual acceptance of the program through initial dubiousness which developed into greater receptiveness of the program as the season progressed. There was an initial sense of caution, relating to stigma associated with the content, and the hesitation of how engagement may reflect upon their role as an athlete.

A coach noted;

"…at first (athletes) were really skeptical about going to our wellbeing people because they thought it was a sign of weakness…”.

This caution was reflected by athletes themselves. One athlete noted;

"…definitely at the start of the season I was kind of still closed off a little bit and then, kind of, you know, going into the season, I was like, you know, probably I should make something out of this, you know, use all the resources I can to help improve my game ”

It was noteworthy that the progression throughout the season reflected a change in approach from players to the wellbeing program. A wellbeing coordinator noted;

“individual buy-in was super by the end of the season”

Coaches and wellbeing coordinators attributed, at least partially, this variation in levels of engagement relative to the impact of COVID-19 lockdowns, which disproportionately affected scheduling of the boys’ program compared to the women's given the timing of the football programs annually. One wellbeing coordinator reported,

"I had about an 85% uptake with the female program and the boys, the … boys, that was that was a lot harder, so probably only 20%, and COVID obviously affected that”

Coaches and wellbeing coordinators also attributed varied engagement in the content itself. This can be seen where one wellbeing coordinator reported,

"those one-on-ones with the girls program was far more engaging, they were honest, they didn't care about what recruiters thought, they confided in me, with that confidentiality they really accepted that, where the boys …. they were still very reserved”.

Another wellbeing coordinator stated,

"from an individual focus, the girls took a lot out of like the individual meetings. The boys, it took time to break down those barriers before they probably were…the girls were a bit more willing to be vulnerable than the boys were”.

This caution was reported to be a barrier preventing some players from experiencing optimal benefits from the program. This can be seen within the report of a wellbeing coordinator who spoke to players’ reluctance to share their experiences,

“… it’s important that they know it’s confidential, and it takes time for them to trust you”

Geographic region was reported to affect engagement with decreased time spent on the program due to the distance from their homes to their training ground. An athlete noted;

"we didn't really have like a large amount of time put on wellbeing kind of thing cause we were rarely at training, cause like we all travel from so far away”.

Some coaches, wellbeing coordinators and players reported a preference for online vs. face-to-face delivery of the program. A number of participants reported finding online delivery of the program to be beneficial, some of which identified as having to travel a significant distance from home to their training ground. In relation to the online delivery of the program, one player stated,

"I found those good, because they were more time efficient for me as well, because I travel so far to training, I kind of just wanted to like get to train and then like, obviously, if I can do wellbeing from home that’s like more convenient as well”.

However, most participants expressed a preference for face-to-face delivery of the program and identified that this method of delivery was more engaging and authentic. An example of this is where a player stated that,

“definitely in-person, in-person helps…”,

Overall, participants engagement gradually increased despite initial hesitation. COVID-19 was an important contextual factor affecting engagement. Some interviewees found the players” engagement to vary throughout the season, with location of the participants (e.g., regional vs. metropolitan) also an important factor affecting engagement. Some reflections indicated gender differences in engagement in the program.

#### 3.1.2 Satisfaction

A sense of connection and support was recurrently identified as significant and prevalent in participants’ experience of the Wellbeing Program. Many perceived their experiences of the program as providing a sense of connection and support which may have contributed to players’ overall satisfaction with the program. This can be seen where one wellbeing coordinator identified that,

“just to have someone completely focus on them, but not just their football or their ability or whatever, but just on them as a whole person”.

When referring to their wellbeing coordinator, a coach reported that they are,

"someone who the players can interact with really easily and have easy conversations. I think that’s been the most beneficial thing”.

These kinds of gestures were recognized by the players and appeared to contribute to the players’ comfortability in approaching the wellbeing coordinators for support, with one player reporting,

"[wellbeing coordinator] was very approachable, I could 100% go to them for anything and everything”,

Generally, players appeared satisfied with the wellbeing coordinators as they provided support and, as reported by players, demonstrated that they had genuine care for the players and their wellbeing, beyond that of the players as athletes, but as individuals.

The individuals involved in the Wellbeing Program expressed many different ways in which they perceived their experience of the program. In terms of satisfaction with the program, coaches, wellbeing coordinators and players identified that certain content delivered within the program was particularly beneficial. Most notably, it was ascertained that the “*high performance period webinar*” was particularly well-received by players.

One wellbeing coordinator reported,

"in terms of curriculum they really liked the session on menstrual cycle and how we can perform best in different parts of your cycle”.

Another wellbeing coordinator reported player satisfaction with this content,

"the biggest one for the girls and the girls bought into was the period in performance part because a lot of them had no knowledge but also the conversations that I had with the girls who were willing to be open”

One player identified that they were satisfied with curriculum including that which focused on content about the body and, content relating to character strengths,

"particularly like those big webinars like learning about our bodies and everything and like how we function as girls as well, like, I think those are like stuff that I really like took into consideration during the season”

The extent to which players had access to their wellbeing coordinator appeared to contribute to their satisfaction and overall acceptability of the program. Most participants reported that the wellbeing coordinator was easily accessible and available for support which contributed to their satisfaction of the program.

One player endorsed this sentiment reporting that,

“it was the open communication that I had with my wellbeing coordinator”

These insights suggest that the participants perceived the wellbeing coordinator to be active, approachable, and easily accessible, which may have contributed to player and staff satisfaction and, overall acceptability of the program.

Participants described variability in satisfaction and thus acceptability of the program due to a sense of there being limited time allocated to delivering the program. An example of this was expressed by one wellbeing coordinator who emphasized that the biggest difficulty in delivering the program was that there was not enough time to do so,

“the biggest challenge is getting time with the players”

A similar perspective was shared by another wellbeing coordinator who stated,

“I think the big thing is we're so time poor in our programs ”

Overall the interviewees reflected a sense of connection and support which contributed to players’ overall satisfaction. Players reported high satisfaction with the wellbeing coordinators as they provided support and care. The extent to which players had access to their wellbeing coordinator appeared to contribute to their satisfaction and overall acceptability of the program. Participants described limited time allocated to delivering the program as a contributing factor to satisfaction with the program.

### 3.2 Feasibility

#### 3.2.1 Participation

Within the theme of feasibility, participants identified that the delivery mode impacted their participation in the program. This can be seen where one coach stated,

"Having so much school online, so many other things conducted online, having to run online workshops, for some of them, no doubt, was just another thing to click into. But I think once we got them in there, they found them really helpful and productive”.

Age was also seen to impact players’ participation in the program through a sense of leadership demosntrated by the older players. This can be seen where some indicated that for participation in the program to increase, it may be valuable for older players to encourage younger players to utilize the wellbeing program. One player noted that,

"I think something not that we would change, but, I think a lot of the older boys should probably try to push other boys to kind of use the wellbeing coordinator as much as they could”.

Similarly, another player noted that as being one of the older players in the team, they adopted a role as a leader in order to encourage other players to participate in the program, noting that,

"I kind of took that as a responsibility role as well, you have to lead the way and hopefully that it helped some of the girls open up too”.

Another player shared a similar sentiment, reporting that,

"by the end of it, everyone was, like, so willing to participate to be part of it, because, yeah, we created a little community, it was really nice and like even the older, the top age girls would set an example and pitch-in and like contribute, and make it feel like a safe space and then the bottom ages would feel comfortable to be a part of it”.

In terms of feasibility and the extent to which the program can be successfully carried out, one method used to measure this was player participation. When discussing their participation, in their accounts, most participants, particularly players, highlighted a preference for one-on-one sessions or smaller group sessions to increase comfortability when sharing. An example of this is where one player noted that,

"I think, just in the one-on-one you feel a little bit more confident just because you know you're, sort of, behind closed doors, and you can sort of speak your mind more, but yeah, I think the one-on-ones are definitely better than the group sessions”.

Another player stated a preference for one-on-one sessions,

"more one-on-one sessions, like maybe set like a weekly catch up or fortnightly catch up just to check in instead of being like a whole team thing because I feel like players might be able to open up a bit more when it’s one-on-one”.

It was identified that a barrier to the feasibility of the program, was players’ self-confidence or lack thereof when participating in the program. Some players recognized that a lack of self-confidence prevented them from getting the most out of the program, particularly when sharing within a group setting. One player reported,

"I was just kind of vulnerable with the wellbeing program, but I think, yeah, just having like confidence issues, that could be a setback for a lot of people”.

Overall, age was a contributing factor to participation. In terms of increasing participation, there was a preference for one-on-one sessions or smaller group sessions to increase likelihood of participation. It also was clear that individual levels factors such as self-confidence and willingness to be vulnerable in the group setting, affected participation in the program.

#### 3.2.2 Retention

In terms of the feasibility in terms of retention in the program, in their accounts, many participants offered suggestions for more practical and beneficial times to deliver the program. Some players expressed a preference for the program to begin in the pre-season,

"I reckon we can probably start it a bit earlier during pre-season as well, because I feel like the THRIVE the individual one-on-one plan that was towards the end of preseason and then like starting in season”.

Another player reported that,

"I think just have like a bigger emphasis on it in pre-season because I think like our body and how it functions… that would probably be like as beneficial as like kicking the footy around for half an hour kind of thing”.

A coach endorsed this sentiment, reporting that,

“we've tried to conduct a lot of it early in the season so that they didn't have fatigue later in the year”.

Overall, the participants reflected a range of opportunities to enhance retention in the program. Broadly, these related to the timing of the program, the relevance of the context relative to the goals of the training program (such as preparing for the season ahead), and recognizing the changing dynamics of the program, and how retention can be enhanced with reference to this (e.g., prior to fatigue which may be increased towards the end of the season).

## 4 Discussion

### 4.1 Overview of findings

This study identified comprehensive insights into the acceptability and feasibility for adolescent program participants involved in the AFL Talent Pathways program. Within the theme of acceptability, participants expressed a general sense of gradual acceptance of the program through initial dubiousness which developed into greater receptiveness of the program as the season progressed. COVID-19 was an important contextual factor in exploring feasibility and acceptability. Some interviewees found the players’ engagement to be low at times throughout the season, with difficulty encouraging players to get involved in the program. Non-metropolitan based players attributed this lack of engagement to decreased time spent on the program due to the geographical distance from their homes to their training ground. Most participants expressed a preference for face-to-face delivery of the program and identified that this method of delivery was more engaging and authentic. Many perceived their experiences of the program as providing a sense of connection and support which may have contributed to players’ overall satisfaction with the program. Generally, players appeared satisfied with the wellbeing coordinators as they provided support and, as reported by players, demonstrated that they had genuine care for the players and their wellbeing. The extent to which players had access to their wellbeing coordinator appeared to contribute to their satisfaction and overall acceptability of the program. Most participants reported that the wellbeing coordinator was easily accessible and available for support which contributed to their satisfaction of the program. Participants described variability in satisfaction and thus acceptability of the program due to a sense of limited time allocated to delivering the program.

Within the theme of feasibility, participants identified that the age of the players impacted their participation in the program. This can be seen where some indicated that for participation in the program to increase, it may be valuable for older players to encourage younger players to utilize the wellbeing program. In terms of feasibility and the extent to which the program can be successfully carried out, one method used to measure this was player participation. When discussing their participation, in their accounts, most participants, particularly players, highlighted a preference for one-on-one sessions or smaller group sessions to increase comfortability when sharing. Implementing smaller groups in order to create an environment where players feel more comfortable to share their experiences may be important. Some players recognized that a lack of self-confidence prevented them from getting the most out of the program, particularly when sharing within a group setting. In terms of the feasibility of the program, in their accounts, many participants offered suggestions for more practical and beneficial times to deliver the program.

### 4.2 Findings in the context of previous literature

There are multiple barriers to adolescent engagement in community-based programs, particularly in the context of peer-to-peer learning and personal skill development (4, 10, 11). The added overlay of the high-performance environment suggests that the young people involved in this program are unique in their experiences, thus further warranting this current study to understand how to best support engagement. Connections to supportive and trusted peers and adult figures is a known enabler to program engagement, and positive development generally (16). Gender differences during the adolescent period are known to impact on program engagement and participation, and these current findings further support the need to integrate such differences from the outset at program development (17). The evidence suggests that the impacts of COVID-19 upon adolescent engagement is multi-faceted, and such impacts are likely to endure through pandemic recovery and beyond. Allemang et al. (18) discussed reduced socializing opportunities, closure of educational institutions, and interrupted engagement in meaningful extracurricular activities are likely to have impacted young people's successful achievement of developmental milestones (18). Considering this, the finding that COVID-19 was identified as a major barrier to participation in our cohort was an expected, but important finding.

The Australian rural and regional settings are characterized by barriers to accessing services such as healthcare and education (19). This is especially so for mental health and wellbeing, to which geographical isolation has been identified as a major contributor to poorer health outcomes observed in rural and regional communities (20). The relevance of geographic isolation to young people's engagement in high performance sport has previously been explored, with both the barriers to accessibility such as physical proximity being noted as an important factor in successful engagement in sport, alongside other non-physical barriers such as socio-economic status (21). An important consideration may be the implications of accessing wellbeing program content when athletes have additional challenges such as long commutes. Indeed, such accessibility issues generate problems with retainment of young athletes in sporting programs generally.

Program scheduling within elite sport is competitive, in that physical skill development has traditionally been the primary focus. It could be considered that these findings highlight the appetite for the integration of wellbeing into high performance environments, however challenges remain in ensuring the prioritisation of such programs. It might be considered that the peer-to-peer relationship strengthening that occurs in the wellbeing programs may be a contributing factor that underpins on-field success. Indeed, there is evidence to suggest that mental wellbeing promotion is viewed as paramount to athletic performance, as identified by athlete cohorts in considering their own lived experience (22). A potential future avenue may be to further strength the team's collective wellbeing. As such, this may further support the goals of the high-performance environment, specifically in terms of team cohesion and capacity building for both the individual athletes alongside the collective whole. This in turn may further support the integration of such programs alongside high performance objectives. Collective wellbeing has gained traction in education and community wellbeing literature and is relative to the broader aims of high performance team sport which hinges on individuals contributing as a collective whole.

### 4.3 Implications of findings

Ultimately this research demonstrated the importance of collaborating with young high performance athletes to understand their needs, and to tailor programs informed by their own lived experience. This study was limited by the small sample size, in that the results may not be reflective of the wider cohort experiences, and thus affects the extent to which findings can be generalised. A larger sample size was not achieved due to recruitment and retention, particularly in the context of the coninciding demands of the high-performance program, combined with the other demands faced by this age group (i.e., completing final year of secondary education). The integration of the wellbeing program in the wider football program is paramount to ensure all athletes are allowed access, so that the wider team club can value and support the mental wellbeing objectives. This approach will contribute to the normalization of such efforts, and in turn overcome initial barriers in engagement and scepticism. Maximizing team building, peer to peer relationships, and collective wellbeing may be a novel opportunity to achieve some of these objectives. For other sporting codes, program expansion might be achieved initially through aligning high performance and wellbeing objectives for young developing athletes, to ensure leadership support and thus appropriate resourcing and sustained efforts. An important practical enabler to implementing such a program is to ensure adequate time is provided to allow the delivery of the program and the integration of wellbeing personnel amongst the competing demands of a high-performance program.

## 5 Conclusions

As identified in this study, and as supported by the wider implementation of science and program design and development evidence body, program success is enhanced with supportive policies and environments alongside individual program components. These findings support this assumption and future research should continue to adopt such complex systems approaches. Although this work was limited by the low sample size in that the themes that emerged may not be reflective of the larger participant group, this study contributes to the small but growing evidence body on young high-performance athletes and the environments in which they train, learn and engage. The novelty of this work is even further enhanced by the focus on athlete mental wellbeing, as opposed to physical health and performance which has been the focus of most literature to date. It is here suggested the incorporation of the above findings, and adopting co-design principles that ensure the voice of the young athletes are central to program development and are integrated into future mental wellbeing programs for young athletes transitioning into elite sport.

## Data Availability

The datasets presented in this article are not readily available because no datasets were generated other than interview data, thus not available for sharing. Requests to access the datasets should be directed to erin.hoare1@deakin.edu.au.
